# Formulaic language identification model based on GCN fusing associated information

**DOI:** 10.7717/peerj-cs.984

**Published:** 2022-06-03

**Authors:** Fanqi Meng, Yujie Zheng, Songbin Bao, Jingdong Wang, Shuaisong Yang

**Affiliations:** 1 School of Computer Science, Northeast Electric Power University, Jilin City, Jilin, China; 2 School of Information Engineering, Guangdong Atv Academy For Performing Arts, Dongguan, Guangdong, China; 3 School of Foreign Language, Northeast Electric Power University, Jilin City, Jilin, China

**Keywords:** Formulaic language, Graph convolutional neural network, Associated information, Mutual information, Dependency syntactic relation

## Abstract

Formulaic language is a general term for ready-made structures in a language. It usually has fixed grammatical structure, stable language expression meaning and specific use context. The use of formulaic language can coordinate sentence generation in the process of writing and communication, and can significantly improve the idiomaticity and logic of machine translation, intelligent question answering and so on. New formulaic language is generated almost every day, and how to accurately identify them is a topic worthy of research. To this end, this article proposes a formulaic language identification model based on GCN fusing associated information. The innovation is that each sentence is constructed into a graph in which the nodes are part-of-speech features and semantic features of the words in the sentence and the edges between nodes are constructed according to mutual information and dependency syntactic relation. On this basis, the graph convolutional neural network is adopted to extract the associated information between words to mine deeper grammatical features. Therefore, it can improve the accuracy of formulaic language identification. The experimental results show that the model in this article is superior to the classical formulaic language identification model in terms of accuracy, recall and F1-score. It lays a foundation for the follow-up research of formulaic language identification tasks.

## Introduction

A formulaic language is a language segment composed of several words to be learned and used as an independent item, and it is a multi-word combination with high frequency (Strobl et al., 2019). It includes a wide variety of categories and functions, such as filler words (*e.g.*, sort of), simple functional phrases (*e.g.*, excuse me), word collocations (*e.g.*, have a break), idioms or proverbs (*e.g.*, spill the beans), and beginning of longer sentences (*e.g.*, there is a growing body of evidence that). Formulaic language identification, also known as multi-word expression identification, refers to finding formulaic language that meets a certain definition from a large amount of corpus, and is also an essential task in natural language processing. The use of formulaic language can not only coordinate sentence generation in the process of writing and communication, improve the accuracy and fluency of language expression, but also have important theoretical and practical significance for computer-assisted language teaching and machine translation (Metin, 2018).

In recent years, the study of formulaic language has been in the ascendant. Scholars have made many research results on formulaic language with the help of corpus technology and computer applications AntConc and AntGram. However, there are some problems such as insufficient recognition standards, low recognition accuracy and efficiency, so recognizing formulaic language efficiently and accurately has become more critical. At present, formulaic language identification methods mainly include the statistics-based method, rule-based method and machine learning method (Hu, Yao & Gao, 2019). The identification method based on statistics and rules depends on the pre-set standards and has poor portability. It cannot effectively recognize many types of formulaic languages in the face of complex text. With the rise of machine learning in the field of natural language processing, some scholars try to use Random Forest (RF), Support Vector Machine (SVM) and other classifiers to identify formulaic language through classification technology. However, this method has high requirements for feature selection (Meng, Cheng & Wang, 2021). It needs to select a feature set that can effectively reflect the characteristics of formulaic language, resulting in its poor generalization ability.

In order to solve the above problems, this article proposes a formulaic language identification model based on GCN fusing associated information. The main technical contributions are summarized as follows:

• This model uses the embedding layer in Torch to generate word embedding vectors as part-of-speech features and uses feature vectors trained by GloVe word vector technology as semantic features. Part-of-speech features and semantic features are represented by Bi-LSTM respectively, and then the two features are fused. The fused feature vector is the basic feature of the formulaic language recognition model, which can fully represent the characteristics of high frequency of co-occurrence and fixed structure of formulaic language.
• This model inputs the associated information (including mutual information and dependency) between words and basic features into the Graph Convolutional Network (GCN) for feature representation. The obtained features can effectively represent the grammatical structure of formulaic language so that the relationship between words can be fully utilized. Finally, the high-order neighbor information between words can be captured.
• The high-order neighbor information is input to the CRF layer for decoding so that the problem of formulaic language recognition is regarded as a sequence labeling problem. This method can obtain the label category of each character, achieve the purpose of recognizing formulaic language, and provide a new idea for solving formulaic language recognition problems.
• We designed three groups of comparative experiments, and the experimental results proved the effectiveness of the proposed model. This model can recognize formulaic language in the text, which lays a foundation for expanding formulaic language corpus in machine translation.

The rest of this article is organized as follows. Section 2 reviews related work. Section 3 describes a formulaic language identification model based on GCN fusing associated information, including basic feature extraction module, feature extraction module fusing associated information and label representation module. Section 4 verifies the effectiveness of the model through experimental analysis. Section 5 summarizes the work of this article and tells the shortcomings and future research directions.

## Related Works

At present, the research on formulaic language is either descriptive research based on the corpus or confirmatory research for language acquisition. There are very little researches related to the recognition of formulaic language. In fact, formulaic language recognition is the basis of applied research on formulaic language (Yu, 2019). However, manually recognizing formulaic language consumes many human resources and material resources, and the recognition efficiency is low. In order to improve efficiency, many scholars have carried out a series of research work on automatic recognition methods.

Formulaic language identification mainly adopts statistics-based method, rule-based method, machine learning method and deep learning method. Statistics-based recognition methods mainly use corpus technology to extract target word bundles from the corpus according to pre-set frequency and distribution standards. In the recognition standard, commonly used corpus parameters include frequency, Mutual Information, *t*-value, dispersion rate, criticality, *etc.* (Li, 2012). The corpus parameter used by Pecina is the phrase frequency, which judges whether several words can form a formulaic language by the frequency of co-occurrence of words (Pecina, 2009). However, if the frequency is used as the only criterion to identify formulaic language, it will ignore some formulaic language composed of low-frequency words with clear textual function, but extract too many phrases composed of high-frequency words without clear textual function. On this basis, the corpus parameters adopted by Li include not only the frequency but also the mutual information value (Lei & Liu, 2018).

Different from the statistical recognition method, the rule-based recognition method sets some part-of-speech and syntactic rules in advance to extract formulaic language (Kirilin, Krauss & Versley, 2016). Iwatsuki, Boudin & Aizawa (2020) believe that a sentence is composed of a formulaic part and an unformulated part, so they proposed to use named entities and dependent structures to remove the unformulated part of the sentence, and the rest is the formulaic part. At the same time, string matching and regular expressions can also be used to identify formulaic language (Durrant & Mathews-Aydnl, 2011). Through multiple linear regression analysis, Wahl (2019) found that the number of rules is positively correlated with the types of recognized formulaic language. The more rules are made, the more types of formulaic language are recognized. However, our rules cannot contain all formulaic language, so the rule-based recognition method is very limited.

With the development of machine learning, Hidden Markov Model (HMM), Support Vector Machine (SVM), Conditional Random Field (CRF), Maximum Entropy (ME) and so on are used by researchers to recognize formulaic language. Abbas et al. (2020) chose to use RF and SVM classifiers to recognize formulaic language through classification. Hyland, Chen, Simpson-Vlach, *etc.* (Hyland, 2008; Chen & Baker, 2010; Simpson-Vlach & Ellis, 2010) proposed to extract N-grams from the corpus, but they can only extract formulaic language with consecutive words. Therefore, Moreau, Alsulaimani & Maldonado (2018) proposed two systems for recognizing formulaic language. CRF-DepTree-categs implements a dependency tree-based method, which aims to use the syntax and semantic relationship between tags; CRF-Seq-nocategs implements a sequence labeling method, which only needs lemma and morphological syntax tags. The experiment found that the latter recognizes the formulaic language with continuous words better, and the former recognizes the formulaic language with discontinuous words better.

With the development of deep learning, Gharbieh, Bhavsar & Cook (2017) tried to use CNN to recognize formulaic language. And they used word2vec training feature vectors. Ashok, Elmasri & Natarajan (2019) compared the role of word2vec and GloVe in recognizing formulaic language on the basis of Gharbieh, and found that GloVe is slightly better in recognizing formulaic language. Taslimipoor & Rohanian (2019) designed a model combining CNN and LSTM. At the same time, in order to deal with the problem of data scarcity in English data sets, firstly, the model is trained on the language with large data, and the learned knowledge is used to predict the tags of formulaic language in English. Subsequently, Taslimipoor, Bahaadini & Kochmar (2020) also designed a system that relies on the pre-trained language model of BERT to achieve semi-supervision of the system through multi-task learning.

The recognition method based on statistics can effectively recognize formulaic language with high frequency of co-occurrence. However, its main problem is that it does not consider the meaning of multi-word combinations and only takes frequency or correlation as the basis of discrimination, so the accuracy of recognition is low. The rule-based recognition method can accurately identify the formulaic language whose form is consistent with the rules defined in the model, which has the problem that the rules are not comprehensive enough. With the development of machine learning, some scholars try to use classifiers such as RF and SVM to recognize formulaic language through classification technology. However, it is necessary to extract features that can represent samples (Meng et al., 2022). The more appropriate the feature selection, the higher the accuracy of recognition. Therefore, feature selection is an urgent problem to be solved (Klyueva, Doucet & Straka, 2017).

To sum up, aiming at the problem of formulaic language recognition, this article proposes a formulaic language identification model based on GCN fusing associated information. This model represents the feature vector through word embedding technology, integrates the associated information that can represent the characteristics of formulaic language, and uses GCN to obtain deeper semantic features. The features obtained in this way are closer to the formulaic language than the features extracted manually in machine learning. Finally, considering the dependency between tags, CRF is used to decode tags to achieve the purpose of recognizing formulaic language. This model not only takes into account the frequency of word co-occurrence, but also considers the dependencies of words in sentences, and achieves good results in formulaic language recognition.

## Model

**Figure 1:**
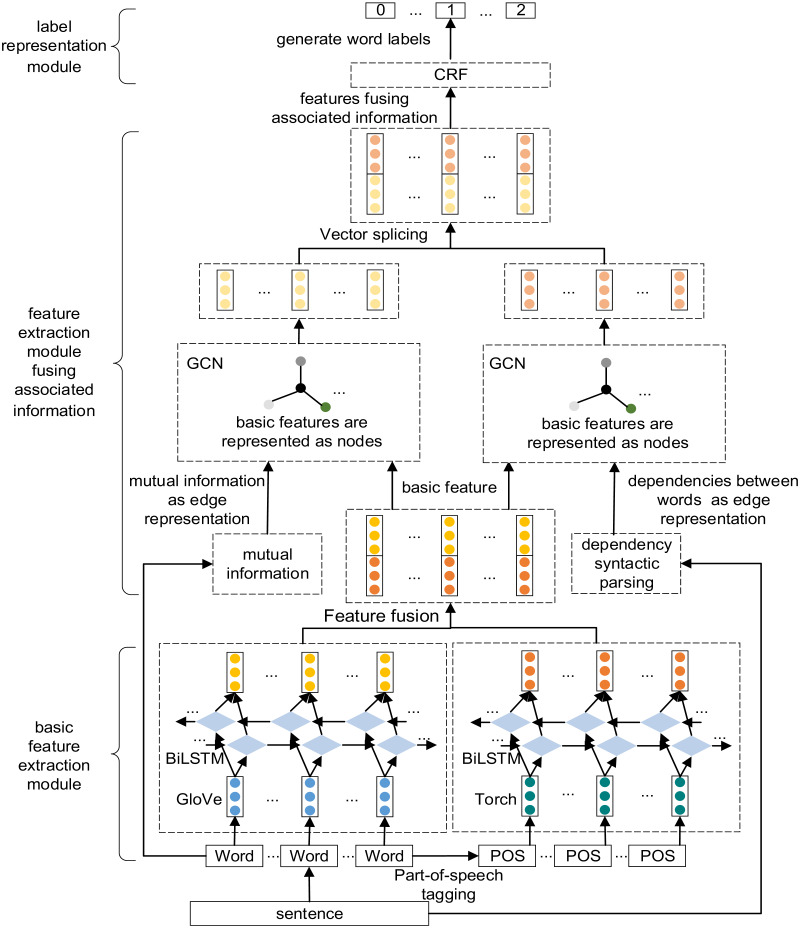
The overall structure of the model.

The model in this article is mainly divided into three parts: basic feature extraction module, feature extraction module fusing associated information and label representation module. Its overall structure is shown in Fig. 1. First, the text’s semantic features and part-of-speech features are extracted and fused through late fusion. The fusion results are used as the basic features of the model. Then the mutual information between words is calculated, the dependency syntactic parsing of sentences is analyzed, and the generated adjacency matrix and basic features are input into GCN for feature representation. Finally, the feature vector is input to the CRF layer for decoding to obtain the label category of each character, so as to obtain the formulaic language.

### Basic feature extraction module

In the process of natural language processing, the computer cannot directly use text data. The text data needs to be expressed as a feature vector, and then the feature vector is used as the model’s input. This article uses the word embedding vector generated by the embedding layer in Torch as the part-of-speech feature, the feature vector trained by GloVe word vector technology as the semantic feature, and the late fusion of the part-of-speech and semantic features as the basic features of the model.

#### Feature selection

The most significant difference between formulaic language and general multi-word expression is that the structure of formulaic language is mostly fixed, which is often in the form of Verb-noun or subject–predicate–object. Therefore, part-of-speech features are regarded as one of the features to identify formulaic language. First, we use the Stanford part-of-speech tagger to analyze the text. The examples of part-of-speech analysis results are shown in Table 1. From the table, it can be found that multi-word units with fixed sentence patterns are more likely to be formulaic language. Then we assign a unique code to each result after part-of-speech tagging, so as to convert the text data into vectors. Finally, we input them into the embedding layer for training to generate word embedding vectors as part-of-speech features.

**Table 1:** The examples of Part-of-speech analysis result.

Formulaic language	Part-of-speech tagging	Sentence structure
X is fundamental to	NN VBZ JJ TO	Subject-Link verb-Predicative Structure
X plays a vital role in the metabolism of	NN VBZ DT JJ NN IN DT NN IN	Subject Verb Object Structure
Several attempts have been made to	JJ NNS VBP VBN VBN TO	Subject Verb Object Structure
In this innovative study, Smith showed that Y	IN DT JJ NN FW FW NN VBD IN NN	Clauses guided by “that”
There were some negative comments about Y	EX VBD DT JJ NNS IN NN	There be...

In addition, for text processing, characters, words, phrases more reflect the lexical information of the text rather than its semantic information, so they can not accurately express the content of the text. For formulaic language, it is a multi-word unit with high frequency and has complete structure, meaning and function, so semantic feature is also an essential feature of formulaic language. This article uses the feature vector trained by GloVe to represent the semantic features of formulaic language.

GloVe’s full name is Global Vectors for Word Representation. It is a word representation tool based on global word frequency statistics. It can express a word as a vector composed of real numbers. These vectors capture some semantic characteristics between words (Pennington, Socher & Manning, 2014). Its implementation is divided into the following three steps:

(1) A co-occurrence matrix *X* is constructed according to the corpus. Each element *X*_*ij*_ in the matrix represents the number of times that word *i* and context word *j* appear together in a context window of a specific size. Generally speaking, the minimum unit of this number is 1, but GloVe doesn’t think so: according to the distance *d* between two words in the context window, it proposes a decreasing weighting function: *decay* = 1/*d* to calculate the weight. That is, the farther the distance, the smaller the weight of the two words in the total count.

(2) Construct the approximate relationship between Word Vector and Co-occurrence Matrix, as shown in Eq. (1).

(1) \begin{eqnarray*}{w}_{i}^{T}\widetilde {{w}_{j}}+{b}_{i}+\widetilde {{b}_{j}}=\log \nolimits ({X}_{ij}).\end{eqnarray*}

Among them, the ${w}_{i}^{T}$ and $\widetilde {{w}_{j}}$ in the above formula are the word vectors we finally need to solve; and *b*_*i*_ and $\widetilde {{b}_{j}}$ are the bias terms of the two-word vectors.

(3) Construct the loss function, as shown in Eq. (2).

(2) \begin{eqnarray*}J=\sum _{i,j=1}^{V}f \left( {X}_{ij} \right) ({w}_{i}^{T}\widetilde {{w}_{j}}+{b}_{i}+\widetilde {{b}_{j}}-\log \nolimits \left( {X}_{ij} \right) )^{2}.\end{eqnarray*}

Among them, $f \left( {X}_{ij} \right) $ is the weight function. Its calculation formula is shown in Eq. (3).

(3) \begin{eqnarray*}f \left( x \right) = \left\{ \begin{array}{@{}l@{}} \displaystyle (x/{x}_{max})^{\alpha } if x\lt {x}_{max} \\ \displaystyle 1 otherwise \end{array} \right. \end{eqnarray*}

Among them, *x*represents the number of co-occurrences, and *x*_*max*_ represents the maximum number of co-occurrences.

#### Feature representation based on Bi-LSTM

Because LSTM is good at capturing the long-distance and long-term dependence of sentence context information, it can better avoid the problem of gradient disappearance and gradient explosion and has higher computational efficiency. Nevertheless, LSTM cannot capture the two-way information of sentences. For the task of formulaic language identification, if the forward information and backward information of sentences are added, the model can learn more semantic information when processing text (Hochreiter & Schmidhuber, 1997). Therefore, we use Bi-LSTM to learn the hidden layer representation of input sequences, hoping to obtain the features of sentences that can contain deeper semantic and syntactic information.

For sentence *s*_*i*_ = [*x*_1_, *x*_2_, …, *x*_*t*_, …, *x*_*n*_], input it into the Bi-LSTM network, we can get the hidden layer representation $ \left\{ {h}_{1},{h}_{2},\ldots ,{h}_{t},\ldots ,{h}_{n} \right\} $ of sentence *s*_*i*_. Each unit obtains the current hidden vector *h*_*t*_ according to the calculation of the previous hidden vector *h*_*t*−1_ and the current input vector *x*_*t*_, and its operation is defined as follows:

(4) \begin{eqnarray*}{i}_{t}& =\sigma \left( {W}_{xi}{x}_{t}+{W}_{hi}{h}_{t-1}+{W}_{ci}{c}_{t-1}+{b}_{i} \right) \end{eqnarray*}

(5) \begin{eqnarray*}{f}_{t}& =\sigma \left( {W}_{xf}{x}_{t}+{W}_{hf}{h}_{t-1}+{W}_{cf}{c}_{t-1}+{b}_{f} \right) \end{eqnarray*}

(6) \begin{eqnarray*}{c}_{t}& ={f}_{t}{c}_{t-1}+{i}_{t}\tan \nolimits h \left( {W}_{xc}{x}_{t}+{W}_{hc}{h}_{t-1}+{b}_{c} \right) \end{eqnarray*}

(7) \begin{eqnarray*}{o}_{t}& =\sigma \left( {W}_{x0}{x}_{t}+{W}_{ho}{h}_{t-1}+{W}_{co}{c}_{t}+{b}_{o} \right) \end{eqnarray*}

(8) \begin{eqnarray*}{h}_{t}& ={o}_{t}\tan \nolimits h \left( {c}_{t} \right) .\end{eqnarray*}

In the formula: *i*_*t*_, *f*_*t*_, *c*_*t*_, *o*_*t*_, *h*_*t*_ are the state of input gate, forget gate, cell state, output gate and hidden layer when the *t*-th text is input; *W* is the parameter of the model; *b* is the bias vector; *σ* is the Sigmoid function; *tanh* is the hyperbolic tangent function.

**Figure 2:**
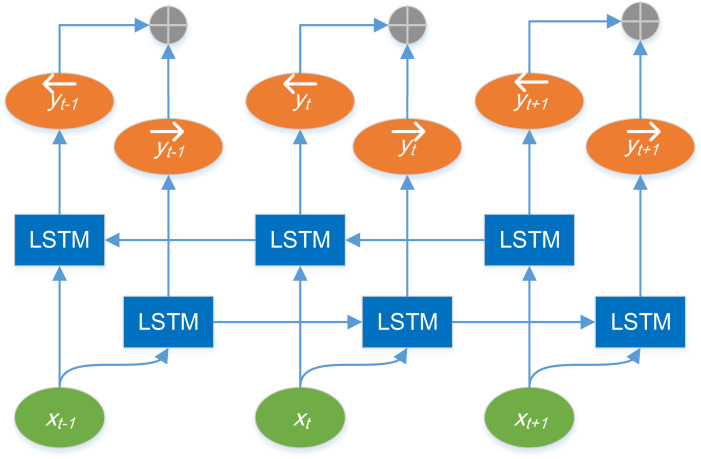
Structure diagram of Bi-LSTM model.

Bi-LSTM model is composed of forward LSTM and reverse LSTM models. The LSTM network of each layer outputs a hidden state information respectively, and the parameters of the model are updated by back propagation. The structure of Bi-LSTM model is shown in Fig. 2.

In Fig. 2, x_t_ represents the input of the network at time *t*, the LSTM in the box is the standard LSTM model, $\vec{{\mathrm{y}}_{\mathrm{t}}}$ is the output of the forward LSTM at time *t*, and ${\mathrm{y}^{\overleftarrow }}_{\mathrm{t}}$ is the output of the reverse LSTM at time *t*.⊕ indicates splicing operation. The output representation of Bi-LSTM at time *t* is defined as ${\mathrm{y}}_{\mathrm{t}}=[{\vec{\mathrm{y}}}_{\mathrm{t}}:{\mathrm{y}^{\overleftarrow }}_{\mathrm{t}}]$, that is, the output at time *t* is directly spliced by the forward output and the reverse output.

#### Feature fusion

**Figure 3:**
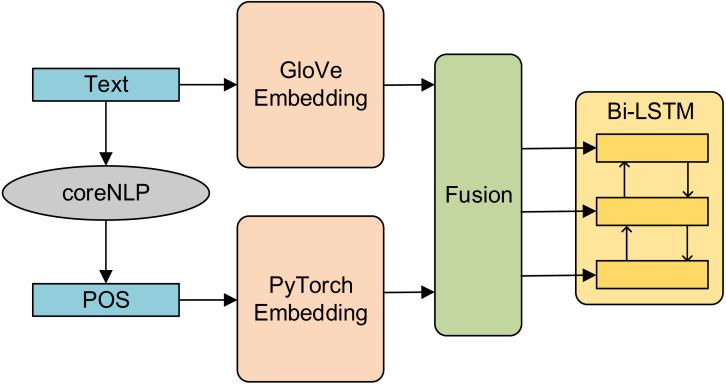
Structure diagram of early fusion model.

Feature fusion includes early fusion and late fusion. Early fusion is to fuse multi-layer features first and then train the model on the fused features (only after complete fusion, unified training). In this article, early fusion is used as a comparative experiment. First, the part-of-speech features and semantic features are fused, and then the fused features are input into Bi-LSTM. The generated results are used as the basic feature vector. The structure diagram is shown in Fig. 3.

**Figure 4:**
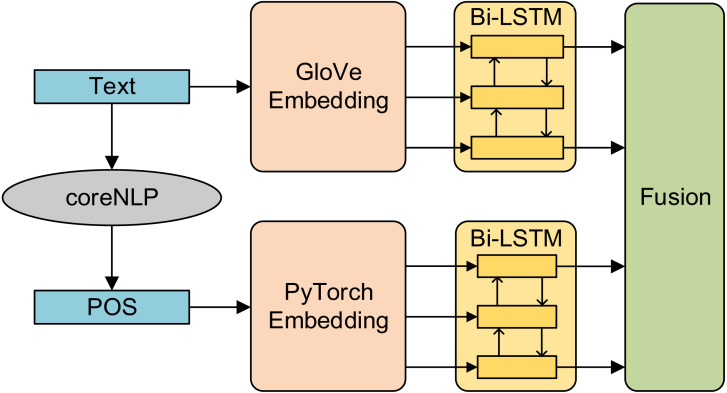
Structure diagram of late fusion model.

Compared with early fusion, late fusion first trains the model with a single feature and then fuses the training results of multiple models. The advantage of late fusion method is that it can flexibly select the model’s results and improve the fault tolerance of the system. The amount of calculation of fusion information is reduced and the real-time performance of the system is improved (Khaleghi, Khamis & Karray, 2013). In this article, the late fusion method is adopted. First, the part-of-speech features and semantic features are input into Bi-LSTM respectively, and then the results of the two models are spliced to form the basic feature vector. The structure diagram is shown in Fig. 4.

### Feature extraction module fusing associated information

The basic feature extraction module uses the word embedding model to train in large-scale text. It can obtain word vectors rich in text semantic features and part-of-speech features, but the syntactic structure information in the text is ignored. As the basis of language understanding, the syntactic structure can effectively represent the grammatical structure of the text and reveal the relationship between the components of the text. For formulaic language, it is a multi-word unit with high frequency of co-occurrence, and several words with high correlations may form formulaic language. Therefore, it is very important to select the features that can represent the relationship between words for identifying formulaic language. Based on this, this article uses the mutual information between words and the dependency syntactic relation of sentences as the associated information of formulaic language.

#### Associated information based on mutual information

Mutual information measures the correlation between two random variables, that is, the amount of information about another random variable contained in one random variable (Zhang & Wang, 2016). The mutual information of two discrete random variables *X* and *Y* is defined as:

(9) \begin{eqnarray*}I \left( X;Y \right) =\sum _{xX}\sum _{yY}p(x,y)\log \nolimits \frac{p(x,y)}{p(x)p(y)} .\end{eqnarray*}

where *p*(*x*, *y*) is the joint probability distribution function of *X* and *Y*, *p*(*x*) and *p*(*y*) are the edge probability distribution functions of *X* and *Y*, respectively. If we want to measure the correlation degree of any two words *x* and *y* in a data set, we can calculate it as follows:

(10) \begin{eqnarray*}I \left( x;y \right) =p \left( x,y \right) \log \nolimits \frac{p \left( x,y \right) }{p \left( x \right) p \left( y \right) } .\end{eqnarray*}

where *p*(*x*) and *p*(*y*) are the probability of independent occurrence of *x* and *y* in the data set. It can be obtained by directly counting the word frequency and dividing it by the total number of words; *p*(*x*, *y*) is the probability that *x* and *y* appear in the data set at the same time. Directly count the number of times they appear at the same time, and then divide it by the number of all unordered pairs. Using mutual information to calculate the relationship between binary words, the higher the mutual information, the higher the correlation between *x* and *y*, and the greater the possibility of forming formulaic language.

#### Associated information based on dependency syntactic parsing

**Figure 5:**
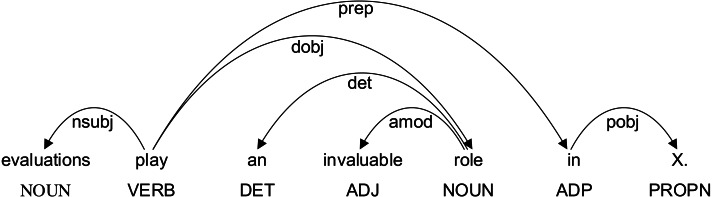
Examples of dependency syntactic relation.

Dependency syntax reveals the dependency relationship and collocation relationship between words in a sentence. One dependency relationship connects two words, one is the core word and the other is the modifier. This relationship is related to the semantic relationship of the sentence. The dependency relationship between words in a sentence includes subject-predicate relationship, verb-object relationship, inter-object relationship, *etc.* The dependency syntactic relation of the sentence “evaluations play an invalid role in X.” is shown in Fig. 5. Among them, “play an invalid role in” is a formulaic language. From the figure, it can be seen that there are complex dependencies between these five words. Therefore, dependency syntactic parsing between words can express the dependency between two words. The closer the relationship is, the more likely it is to form a formulaic language.

#### Feature representation based on GCN

In the formulaic language identification using dependency syntactic relation, the existing studies mostly use the dependency syntax in the text to construct rules, extract features, and then input them into the classifier to recognize formulaic language through classification. Although this method can achieve certain results, the nonlinear semantic relationship between components in sentences has not been learned and utilized. Spatially, the relationship between words can be represented by a graph through mutual information and dependency parsing. The GCN is used to process the correlation information, so that the nonlinear semantic relationship between words in the sentence can be extracted.

**Figure 6:**
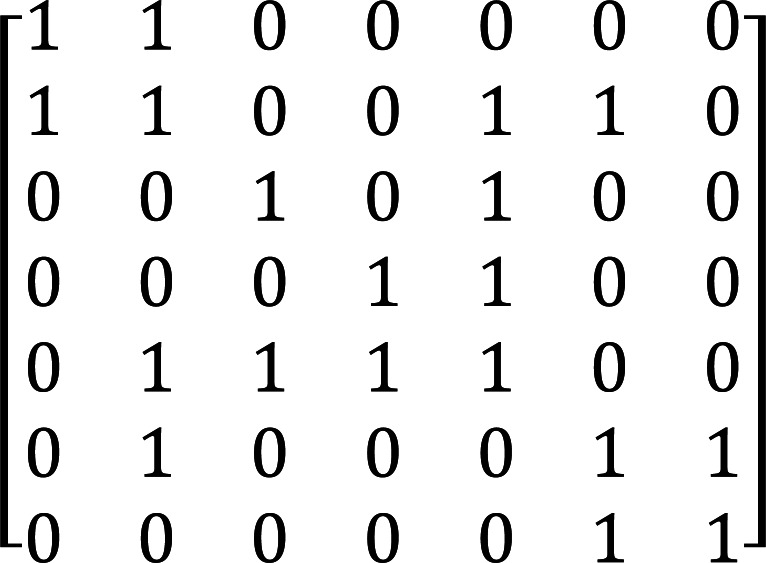
Adjacency matrix constructed based on dependency syntactic parsing.

In the feature representation based on GCN, the mutual information and dependency syntactic relation between words are used to determine the word connection relationship, and the basic features of words are represented as nodes. For the GCN with mutual information as input, firstly, the corpus is used as the data set to calculate the mutual information value between words, the word is used as the node, the mutual information value between nodes is used as the representation of the edge. The element *a*_*ij*_ in its adjacency matrix *AɛR*^*N*∗*N*^represents the mutual information value between the *i*th node and the *j*th node in the graph. For the GCN with dependency syntactic relation as input, firstly, the dependency syntax of sentences is analyzed, with words as nodes and the dependency between words as the representation of edges. The element *a*_*ij*_ in its adjacency matrix *AɛR*^*N*∗*N*^represents the dependency between the *i*th node and the *j*th node in the graph. If there is a dependency between the two nodes, the *a*_*ij*_ is 1, otherwise it is 0. For example, in the example of dependency syntactic parsing “evaluations play an invaluable role in X.”, the adjacency matrix A constructed based on dependency syntactic parsing is shown in Fig. 6.

**Figure 7:**
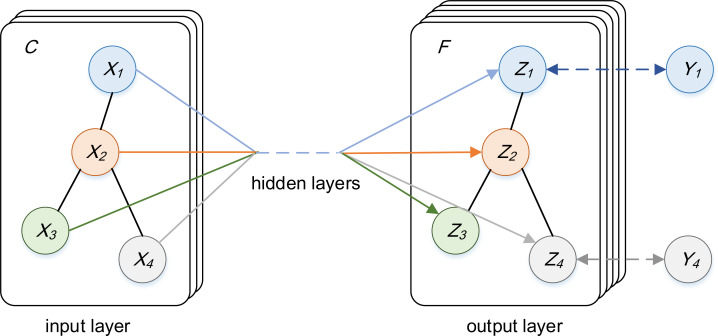
The structure diagram of GCN.

In order to directly conduct deep learning modeling on graph data, the specific method adopts a variant model of convolution neural network-Graph Convolution Neural Network proposed by Kipf & Welling (2016), and the structure is shown in Fig. 7. The channels input by GCN is *C*, that is, the feature vector dimension of each node *X*_*i*_ is *C*, the channels output by GCN is *F*, that is, the feature vector dimension of each node *Z*_*i*_ is *F*, and finally the label *Y*_*i*_ of the node is predicted by the feature of the node.

Specifically, given a graph *G* = (*V*, *E*), *V* is a vertex set containing *N* nodes, *E* is an edge set including self-loop edges (that is, each vertex is connected to itself). The feature information of graph *G* = (*V*, *E*)can be represented by Laplace matrix, as shown in Eq. (11).

(11) \begin{eqnarray*}L=D-A.\end{eqnarray*}

Or use the symmetric normalized Laplasse matrix, as shown in Eq. (12):

(12) \begin{eqnarray*}{L}^{sys}={I}_{N}-{D}^{-1/2}A{D}^{-1/2}.\end{eqnarray*}

where *A* is the adjacency matrix of the graph, *I*_*N*_ is the *n*-order identity matrix; *D* = *diag*(*d*) is the degree matrix of the vertex, as shown in Eq. (13).

(13) \begin{eqnarray*}{D}^{ii}=\sum _{j}{A}_{ij}.\end{eqnarray*}

Based on the Fourier transform of graph, the graph convolution formula can be expressed as Eq. (14):

(14) \begin{eqnarray*}g\mathrm{ \ast }x=U[ \left( {U}^{T}g \right) \cdot ({U}^{T}x)].\end{eqnarray*}

In the formula, *x* is the basic feature vector of the node; *g* is convolution kernel; *U* is the eigenvector matrix of Laplace matrix *L*.

In order to reduce the amount of calculation, scholars used Chebyshev polynomials to simplify the graph convolution formula in 2017. Finally, the layered propagation formula of graph convolution can be expressed as Eq. (15):

(15) \begin{eqnarray*}{X}^{(l+1)}=\sigma [{\tilde {D}}^{-1/2}\tilde {A}{\tilde {D}}^{-1/2}{X}^{ \left( l \right) }{W}^{ \left( l \right) }].\end{eqnarray*}

In the formula, $\tilde {A}=A+{I}_{N}$, $\tilde {D}=\sum \tilde {A}$; *σ* is the activation function; *W* is the weight matrix to be trained.

### Label representation module

Conditional Random Field (CRF) is a statistical-based learning model first proposed by Lafferty, McCallum & Pereira (2001). Through the calculation and statistics of known features, the conditional probability of unknown features can be inferred. Based on the maximum entropy model and hidden Markov model, this model is mainly used for sequence tagging. It has achieved good recognition results in named entity recognition, syntactic analysis and part-of-speech tagging in the field of natural language processing. In the model of this article, a conditional random field is added at the end of the model to predict the label category, so as to identify formulaic language in the sentence.

For an undirected graph *G* = (*V*, *E*), *Y* = *Y*_*v*_|*v* ∈ *V* is a set of random variables *Y*_*v*_ indexed by node *V* in *G*. For the conditions that X has been given, if each random variable *Y*_*v*_ obeys the Markov property, the probability of *Y*_*v*_ can be expressed as Eq. (16), then (*X*, *Y*) is established as a conditional random field.

(16) \begin{eqnarray*}P \left( {Y}_{v}{|}X,{Y}_{u},u\not = v \right) =P({Y}_{v}{|}X,{Y}_{u},u\sim v).\end{eqnarray*}

Among them, *u* ∼ *v* represents that node *u* and node *v* are adjacent in the undirected graph *G*.

**Figure 8:**
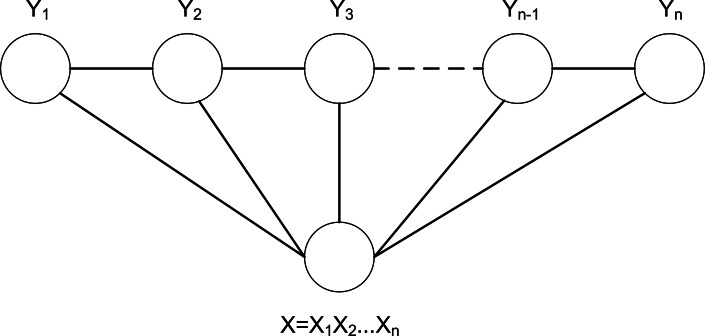
The structure diagram of linear-chain CRF.

When constructing CRF model, the linear-chain CRF commonly used in sequence labeling problem is selected. The structure diagram of linear-chain CRF is shown in Fig. 8. Among them, the input observation sequence is represented by *X*, and *X* is used as a precondition. *P*(*y*|*x*) is defined as a joint probability distribution, and the maximum value among them is selected as the corresponding sequence label (Ren, Zhang & Zhang, 2019).

## Experimental Setup and Analysis

In order to recognize formulaic language in the text more accurately, this article proposes a formulaic language identification model based on GCN fusing associated information. It sets up three groups of comparative experiments. There are two purposes of the experiment. One is to select the optimal feature combination and complete the construction of the recognition model through ablation experiment and network layer comparison experiment. The second is to verify the effectiveness of the proposed model by comparing with the existing recognition models.

### Experimental data and labeling strategy

We downloaded 20 articles in the computer field from the Web of Science, preprocessed the text, removed references, pictures, formulas, *etc.*, and then segmented the sentences. A total of 1889 sentences were obtained as the data set. We take the formulaic language in the formulaic language corpus of Manchester University as the standard. Three professors of English major are invited to extract the formulaic language in the sentences. There are 2,563 formulaic languages in total, and 1,034 are left after the reprocessing. Then we tag the sentence. The tagging strategy adopts the BIO tagging method, ‘B’ means that the word is the starting position of the formulaic language, ‘I’ means that the word is the middle position of the formulaic language, and ‘O’ means that it does not belong to the formulaic language.

### Evaluating indicator

In this article, the PRF index is used to evaluate the experimental results of formulaic language recognition. P represents the accuracy of formulaic language recognition; R represents the proportion of the correct number of recognized formulaic languages in the total number of formulaic languages in the corpus, which is called recall; F-score is the synthesis of P and R as the comprehensive index to evaluate the effect of formulaic language recognition. The three formulas correspond to the Eqs. (17), (18) and (19):

(17) \begin{eqnarray*}P& = \frac{{N}_{m}}{{N}_{total}} \times 100\text{%}\end{eqnarray*}

(18) \begin{eqnarray*}R& = \frac{{N}_{m}}{{N}_{correct}} \times 100\text{%}\end{eqnarray*}

(19) \begin{eqnarray*}F& = \frac{2\times P\times R}{P+R} .\end{eqnarray*}

Among them, *N*_*m*_ represents the number of correctly recognized formulaic language, *N*_*total*_ represents the total number of recognized formulaic language, and *N*_*correct*_ represents the total number of manually marked formulaic language.

### Parameter settings

In the model, semantic features are 300-dimensional word vectors trained by Glove, part-of-speech features are 300-dimensional word vectors trained by the embedding layer. The basic feature extraction module uses Bi-LSTM to extract features, each layer has 128 neurons. The feature extraction module fusing associated information uses GCN to extract features, including two GCNs. The GCN based on mutual information has two layers of graph convolution, and the GCN based on dependency syntactic parsing has three layers of graph convolution. The output of the GCN layer is set to 64.

During model training, Adam optimizer is selected as the optimization algorithm, the learning rate is set to 0.001, and the decay ratio is set to 0.9.

### Ablation experiment

In order to better verify the effect of formulaic language identification model based on GCN fusing associated information, seven comparative experiments were conducted to determine which feature is more important for formulaic language identification by setting ablation experiments. The specific methods are as follows:

• Before_Bi-LSTM: The part-of-speech features generated by PyTorch word embedding and the semantic features generated by GloVe word embedding are fused by early fusion. The fused feature vector is input into Bi-LSTM to extract the context semantic relationship. Finally, the features generated by Bi-LSTM are input into CRF to complete the recognition of formulaic language.
• Before_CNN: The part-of-speech features generated by PyTorch word embedding and the semantic features generated by GloVe word embedding are fused by early fusion. The fused feature vector is input into CNN. The size of convolution kernel is 3 * 3, and a total of two layers of CNN are set. Finally, the features generated by CNN are input into CRF to complete the recognition of formulaic language.
• After_Bi-LSTM: The part-of-speech features generated by PyTorch word embedding and the semantic features generated by GloVe word embedding are fused by late fusion. That is, the two feature vectors are input into Bi-LSTM respectively, and then the processed vectors are fused. Finally, the fused features are input into CRF to complete the recognition of formulaic language.
• After_Bi-LSTM_CNN: Based on After_Bi-LSTM, this method adds a layer of CNN between Bi-LSTM and CRF. The size of convolution kernel is 3 * 3, and a total of two layers of CNN are set.
• Bi-LSTM_DS_GCN: The part-of-speech features generated by PyTorch word embedding and the semantic features generated by GloVe word embedding are fused by late fusion. The fused feature vector is used as the basic feature and the matrix generated by dependency syntactic relation is used as the associated feature, which are input into GCN for feature representation. Finally, the features generated by GCN are input into CRF to complete the recognition of formulaic language.
• Bi-LSTM_MI_GCN: The difference from Bi-LSTM_DS_GCN is that the matrix generated by dependency syntactic relation is replaced with the matrix generated by the mutual information between words as the associated feature.
• Bi-LSTM_MI_DS_GCN: This is the model proposed in this article. The late fused part-of-speech features and semantic features are used as the basic features and the matrices generated according to mutual information and dependency syntactic relation are used as the correlation information. The basic features and correlation information are input into GCN. Finally, the features are decoded through CRF.

**Figure 9:**
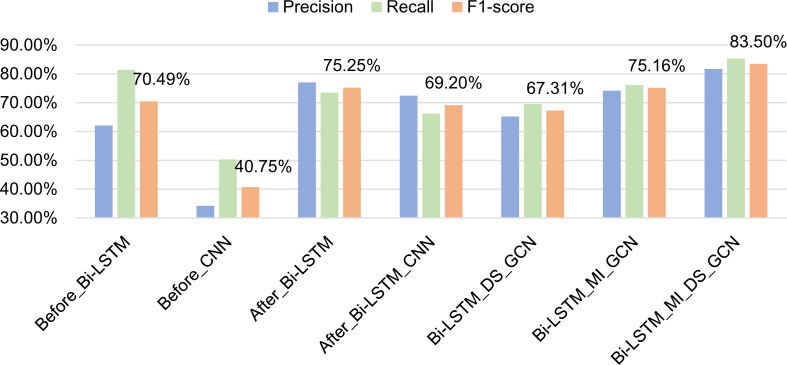
Comparison of experimental results of ablation experiment.

Seven methods are tested on the data set, and the experimental results are shown in Table 2 and Fig. 9. In Table 2, train_time represents the time required to train 100 pieces of data, and test_time represents the time required to test 100 pieces of data.

Analysis of experimental results:

(1) The difference between Experiment 1 and Experiment 2 lies in the comparison between Bi-LSTM and CNN. The experimental results show that Bi-LSTM is much better than CNN in feature extraction. Because the identification of formulaic language is a typical sequence labeling problem, Bi-LSTM can capture the long-distance and long-term dependence of sentence context information and can capture the two-way information of sentences. However, CNN cannot capture the long-distance dependence information well, so it is better to use Bi-LSTM in formulaic language recognition. It is worth noting that the Recall in the result of using CNN to extract features is relatively high, indicating that it can recognize more formulaic languages. However, it can also recognize many non-formulaic languages, so the Precision is not very high.

**Table 2:** Comparison of experimental results of ablation experiment.

Number	Method	Precision	Recall	F1-score	Train_time	Test_time
1	Before_Bi-LSTM	62.12	81.46	70.49	9.40775	3.83115
2	Before_CNN	34.23	50.33	40.75	11.24228	5.06429
3	After_Bi-LSTM	77.08	73.51	75.25	11.96498	5.16604
4	After_Bi-LSTM_CNN	72.46	66.23	69.20	22.86475	8.34037
5	Bi-LSTM_DS_GCN	65.22	69.54	67.31	12.20419	4.36681
6	Bi-LSTM_MI_GCN	74.19	76.16	75.16	11.19024	3.63964
7	Bi-LSTM_MI_DS_GCN	**81.71**	**85.37**	**83.50**	13.55199	5.35704

(2) The difference between Experiment 1 and Experiment 3 is that the way of feature fusion is different. Experiment 1 adopts early fusion and Experiment 3 adopts late fusion. The experimental results show that the late fusion method has higher Precision and the early fusion method has higher Recall. The main reason is that the early fusion method can recognize more results when recognizing formulaic languages, but it can also recognize many non-formulaic languages, so its Precision is low. The features of the late fusion method are more accurate, and the recognition results are not as much as those of the early fusion, but the formulaic languages can be recognized more accurately. According to the F1-scores of the two methods, the effect of late fusion is better than that of early fusion.

(3) Experiment 4 adds CNN layer based on Experiment 3, but the results after adding CNN are not as good as the previous results. The main reason is that CNN captures local correlations and each layer of CNN has a fixed span. Naturally, this layer can only model limited distance information. In Experiment 4, Bi-LSTM has obtained the long-distance dependency of the context, and then CNN is added after Bi-LSTM to deeply abstract the features, some correct formulaic languages are filtered out, resulting in a significant decline in Precision and Recall.

(4) Experiment 5 adds feature extraction based on GCN fusing dependency syntactic parsing on the basis of Experiment 3. The experimental results showed that after adding dependency syntactic features, the Recall and Precision are reduced. Because dependency syntactic relation mainly focuses on the dependency between two words in a sentence, it is easy to cause that the extracted word string does not belong to formulaic language, so the recognition effect is not good.

Experiment 6 adds feature extraction based on GCN fusing mutual information on the basis of Experiment 3. The experimental results show that after adding the mutual information features, the Recall increases, indicating that the number of correctly recognized formulaic language is more. However, due to the reduction of Precision, F1-socre is not improved. At the same time, compared with experiment 5, it shows that mutual information features can better represent the characteristics of formulaic language than dependency syntactic features.

(5) Experiment 7 (the model proposed in this article) inputs the dependency syntactic features and mutual information features into GCN for feature extraction. The experimental results show that although the independent dependency syntax features (Experiment 5) do not show good results, after combining the two, the Precision. Recall have increased significantly. The reason is that the features of dependency syntactic parsing and mutual information complement each other in the identification of formulaic language to achieve efficient extraction of formulaic language. It also shows that dependency syntax features and mutual information features are important features to measure formulaic language.

(6) From the train_time and test_time in Table 2, it can be seen that even though the number of features fused by the model in this article increases, the training time does not increase much, and the training time is less than the method using CNN. At the same time, the time for the model to test 100 pieces of data is only 3.7% higher than that of experiment 3 (the experiment with the highest F1-score among other experiments). This shows that the model in this article does not reduce the efficiency of formulaic language identification while improving the Precision and Recall. It is an efficient and accurate model for the identification of formulaic language.

In summary, through ablation experiments, the results verify the effect of formulaic language identification model based on GCN fusing associated information. That is, through the late fused part-of-speech features and semantic features as the basic features, dependency syntactic relation and mutual information as the associated information, the combined model can reduce the errors of a single model and enhance their advantages.

### Comparative experiment of different network layers

Because the model in this article involves two GCNs, one is GCN based on dependency syntactic parsing, and the other is GCN based on mutual information. Therefore, when selecting the number of layers of GCN, we use Experiment 5 and Experiment 6 in ablation experiment to do two groups of comparative experiments and select the optimal number of network layers by comparing the experimental results.

**Figure 10:**
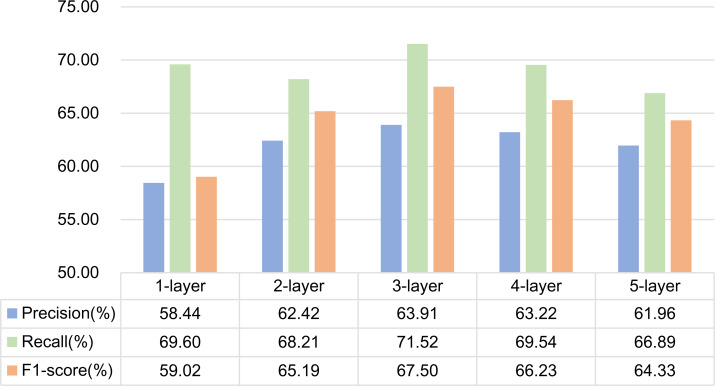
Influence of different network layers on GCN based on dependency parsing.

**Figure 11:**
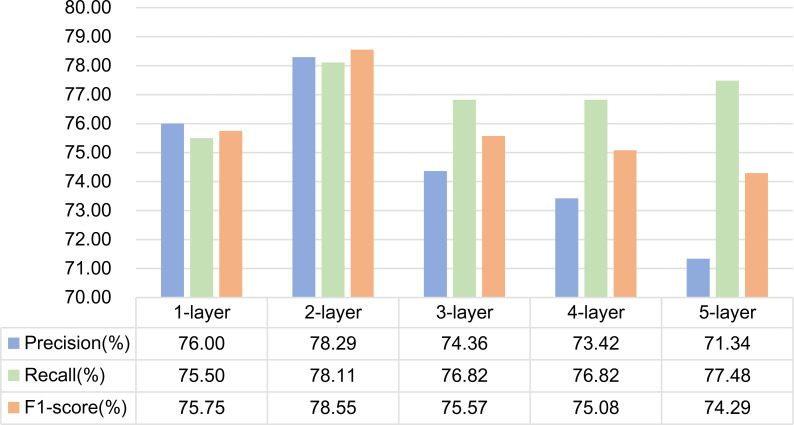
Influence of different network layers on GCN based on mutual information.

(1) Experiment 5 is a GCN structure based on dependency syntactic parsing. Experiments are carried out by setting 1, 2, 3, 4 and 5-layer graph convolution respectively. The experimental results are shown in Fig. 10. As can be seen from the figure, the effect of using 3-layer graph convolution is the best for dependency syntactic parsing.

(2) Experiment 6 is a GCN structure based on mutual information. Experiments are carried out by setting 1, 2, 3, 4 and 5-layer graph convolution respectively. The experimental results are shown in Fig. 11. As can be seen from the figure, the effect of using 2-layer graph convolution is the best for mutual information.

Analysis of experimental results:

It is generally believed that increasing the number of network layers can reduce the error and improve the accuracy, but it will also complicate the network, thus increasing the training time of the network and the tendency of over fitting. Therefore, the number of network layers required by different features is different. In this article, dependency syntactic parsing only uses 0 or 1 to describe the relationship between two words, which is not good enough for the interpretation of formulaic language, so it needs three-layer graph convolution to extract better features. Mutual information can better represent the characteristics of formulaic language, so two-layer graph convolution can extract better features.

From the above analysis, it can be seen that in formulaic language identification model based on GCN fusing associated information, the layers of the two GCNs are set to 2 and 3 layers respectively.

### Comparative experiments of different models

In order to verify the effectiveness of the model proposed in this article, the CNN_Bi-LSTM_CRF model in literature (Gao, Peng & Bai, 2020) and the Bi-LSTM_CRF model in literature (Berk, Erden & Güngör, 2018) are selected for comparison.

• CNN_Bi-LSTM_CRF: This article performs the task of named entity recognition. Because formulaic language recognition and named entity recognition are similar, and this model performs well in the field of named entity recognition, this model is used as a comparative experiment. The word vector is trained with word2vec, and the word vector of the text data obtained after word2vec training is spliced to generate the word vector matrix, which is then used as the input of CNN convolution layer. The CNN module extracts the spatial feature information of the text through convolution and the collection of vector matrix. Then, the results are input into Bi-LSTM for forward and backward training. Finally, the vector with sentence feature information is put into CRF for decoding and prediction to obtain the final sequence.
• Bi-LSTM_CRF: This article describes the Deep-BGT system that participated in the PARSEME shared task 2018 to automatic identification of verbal multi-word expressions (VMWEs). Author uses Bi-LSTM model with a CRF layer on top. The input layer includes word vectors generated by fastText word embedding technology, POS and dependencies. Each input vector is represented as splicing these three features, similar to early fusion technology. Because formulaic language is also a kind of multi-word expression, the combination of Bi-LSTM and CRF is the mainstream method in the field of multi-word expression recognition, so this model is used as a comparative experiment.

**Figure 12:**
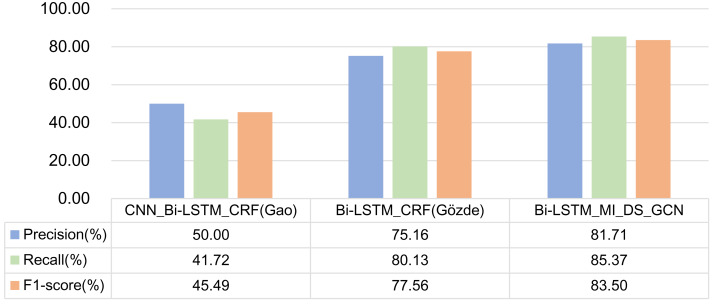
Comparison of experimental results of three models.

The experimental results of the above two models and the model proposed in this article on formulaic language recognition are shown in Fig. 12.

Analysis of experimental results:

(1) As the CNN_Bi-LSTM_CRF model is the primary method in named entity recognition, the experimental results show that this model does not perform well in the task of formulaic language recognition. It can be seen that in different tasks, although the two tasks are very similar, we should also start from the essence of the object in the task, mine the characteristics that can represent the research object, and design a unique model. At the same time, through the CNN_Bi-LSTM_CRF model and Experiments 2 and 4 in the previous section, it can be found that the effect will not get better after adding CNN to the model. Hence, CNN is not suitable for formulaic language identification task.

(2) Bi-LSTM_CRF model is used to identify multi-word expressions. Compared with Experiment 1 in the previous section, the difference lies in the different input features. The input features of Experiment 1 are part-of-speech features and GloVe word embedding features, while Bi-LSTM_CRF model inputs part-of-speech features, fastText word embedding features and dependency syntactic relation. The experimental results show that the F1 -score of Bi-LSTM_CRF model is higher. However, the Recall of Experiment 1 is higher, indicating that dependency syntactic relation is conducive to recognizing formulaic language.

At the same time, in comparison with the model in this article, the main difference is that the Bi-LSTM_CRF model only constructs the dependency syntactic relation into a simple feature vector, splices it with other features and then trains them. The model in this article constructs the graph structure through the syntactic dependency tree and then extracts the features through GCN. The advantage of GCN is that it can gather the information on all edges and points. Thus, the boundary ambiguity between words is eliminated, and any two non-adjacent nodes in the graph are each other’s second-order neighbors. They can receive each other’s non-local information through two-node updates. The aggregated features in this method can more accurately represent formulaic language, so the effect of identifying formulaic language is better.

## Error analysis

In order to make an in-depth analysis of the shortcomings of this model, we investigated the examples of formulaic language recognition errors under the best experimental results and selected three representative sentences for analysis. The specific contents are shown in Table 3.

**Table 3:** Examples of formulaic language recognition errors.

Text	Formulaic language	Output
It is a nontrivial task since we do not have the ground truth labels to decide the adjustment.	It is a nontrivial task since	It is a nontrivial task
In section 7 we compare these two variants against the regular hash function as well as to other leading hashing schemes found in the literature.	As well as	As well as to
We therefore resort to an approximation of the process which has a negligible impact on the overall precision but greatly reduces run time.	Has a negligible impact on	Resort to; has a negligible impact on

It can be seen from the table that the reason for the recognition error in the first example is that the word ‘since’ is not recognized. The reason for the recognition error in the second example is that the word ‘to’ is recognized. The reason for the recognition error in the third example is that ‘restore to’ is also regarded as a formulaic language. For the first two examples, in fact, the model’s positioning in the sentence is very accurate. However, due to the uncertainty of manual labeling, fixed evaluation indicators and other factors, it can only be considered that this word string recognition is wrong. If the recognition effect is viewed from the perspective of word coverage, it must be higher than the existing evaluation indicators.

In the third example, although ‘restore to’ is a non-formulaic language, it contains many characteristics of formulaic language, resulting in the model mistakenly considering it as formulaic language. This kind of word string usually contains many characteristics of formulaic language, such as Verb + preposition and other structures. However, it may not have a specific function and meaning in the fields of scientific and technological literature writing, so they are not formulaic languages. This situation is a problem we should think about and solve in the future.

## Conclusions

In this article, a formulaic language identification model based on GCN fusing associated information is proposed. The part-of-speech features and semantic features fused through late fusion are taken as the basic features. Then the two associated information of mutual information and dependency syntactic relation are input into GCN together with the basic features for feature representation. This combined representation can capture the syntactic and semantic structure of multi semantic web. It can also conduct a more in-depth downstream semantic analysis. Finally, we input the feature vector output from GCN layer to CRF layer for decoding, obtain the label category of each character, and get the formulaic language. Several groups of comparative experiments show that compared with the existing models, the proposed model can improve the accuracy of formulaic language identification and verify the model’s effectiveness. In addition, it should be noted that the formulaic language identification model proposed in this article can obtain strong identification performance with only a small proportion of labeled text. For the identification of other tasks, it has a certain universal reference value. However, this model still has some shortcomings in analyzing the fuzziness and fuzzy boundary of some formulaic languages. The word strings described by facts in sentences cannot be understood and learned by the model, which needs further consideration in future research.

## Supplemental Information

10.7717/peerj-cs.984/supp-1 Supplemental Information 1 Data and Code

## Additional Information and Declarations

### Data Availability

The following information was supplied regarding data availability: The code and data is available in the Supplemental Files and at Github: https://github.com/access1-sys/Syntax_analysis.
